# Brucellosis Seroprevalence, Analysis of Risk Factors, and Comparison of Test Methods Used in Diagnosis in a Tertiary Hospital in Kahramanmaraş

**DOI:** 10.3390/tropicalmed11030085

**Published:** 2026-03-21

**Authors:** Özlem Kirişci, Zerife Orhan

**Affiliations:** 1 Department of Medical Microbiology, Faculty of Medicine, Kahramanmaraş Sütçü İmam University, 46040 Kahramanmaraş, Türkiye; dr_ozlemgitmisoglu@hotmail.com; 2 Department of Medical Services and Techniques, Vocational School of Health Services, Kahramanmaraş Sütçü İmam University, 46040 Kahramanmaraş, Türkiye

**Keywords:** brucellosis, seroprevalence, risk factors, serological tests, Türkiye

## Abstract

(1) Brucellosis is a zoonotic infection that remains a significant public health concern in endemic regions. This study aimed to determine the seroprevalence of brucellosis in a tertiary care hospital, analyze associated risk factors, and evaluate the diagnostic performance of commonly used serological tests. (2) The study was based on the serological test results of 24,545 samples collected between 2020 and 2023. Rose Bengal, standard tube agglutination, and Brucellacapt tests were used for the diagnosis of brucellosis. Data were analyzed according to age, sex, clinical department, and seasonal distribution using SPSS version 25.0. (3) Overall, 367 cases (1.5%) tested positive. When the 367 seropositive cases were evaluated by year, the annual distribution showed a declining trend, decreasing from 2.5% in 2020 to 1.2% in 2023. Among the positive cases, 57.8% were female, and 36% were aged between 41 and 64 years. The infectious diseases department had the highest positivity rate (37.1%). Brucellacapt showed the highest positivity rate (90.2%), followed by Rose Bengal (76.2%). The highest monthly positivity rate was observed in October (11.4%), and seasonally in autumn (31.3%). (4) The Brucellacapt test has demonstrated high sensitivity and serves as a valuable supplementary diagnostic tool in the evaluation of brucellosis. However, its low specificity underscores the necessity for careful interpretation of positive results and supports its use in conjunction with other serological tests to enhance diagnostic accuracy. Considering seasonal and departmental variations, a combined testing approach may improve overall diagnostic accuracy.

## 1. Introduction

Brucellosis is a zoonotic infectious disease caused by Gram-negative bacteria of the *Brucella* genus and remains a significant global health problem, particularly in regions where agriculture and animal husbandry are widespread [1]. The Food and Agriculture Organization of the United Nations, the World Health Organization (WHO), and the World Organisation for Animal Health classify brucellosis as one of the most neglected zoonotic diseases worldwide. The disease can be transmitted to humans through direct contact with infected animals, consumption of raw or unpasteurized milk and other dairy products, or accidental ingestion or inhalation of contaminated materials [2].

Brucellosis is prevalent in more than 170 countries, particularly in the Mediterranean region, Asia, and the Americas [3]. More than 500,000 new cases are reported annually, with incidence rates exceeding 10 per 100,000 population in some regions [4]. In Türkiye, the disease is endemic, with an incidence of 7.99 per 100,000 reported in 2017 [5].

According to a comprehensive systematic review and meta-analysis including 92 studies conducted between 1993 and 2024, the pooled prevalence of human brucellosis in Middle Eastern countries was 20.45% (95% CI: 17.85–23.19%), indicating a substantial disease burden in the region. Country-specific prevalence rates varied widely, ranging from approximately 15% in Türkiye to over 50% in Kuwait and Qatar, reflecting heterogeneity related to population characteristics, diagnostic methods, and exposure risks [6]. In Türkiye, a meta-analysis conducted between 1999 and 2021 estimated the overall seroprevalence of human brucellosis at 4.5%, increasing to up to 8% in rural areas; notably higher prevalence rates of approximately 13% have been reported in the Central East Anatolia region [5].

If left untreated, the disease may become chronic and involve multiple organs, necessitating early and accurate diagnosis [7]. Brucellosis is difficult to diagnose because of its non-specific symptoms and the challenges associated with bacterial isolation [8], which may be influenced by disease stage, bacterial load, incubation period, and prior antibiotic use. Since blood cultures are not performed in all healthcare facilities, carry infection risks, and are time-consuming, serological tests are more commonly used [9,10,11]. However, these tests may yield ambiguous and difficult-to-interpret results [12].

In 2006, the WHO recommended the Rose Bengal test (RBT) as a sensitive and rapid screening test, followed by confirmation with more specific methods such as the standard tube agglutination test (STA), ELISA, or the microagglutination test [13]. STA and the *Brucella* agglutination test (with Coombs antiserum) are widely used laboratory methods for diagnosis [10]. The Brucellacapt test, as an alternative to the STA and Coombs tests, is particularly useful in the diagnosis of complicated and chronic cases. It offers advantages due to its rapid application and its ability to demonstrate a prompt decline in antibody titers following successful treatment [11,14].

This study aimed to determine the seroprevalence of brucellosis in a tertiary hospital in Kahramanmaraş, identify patient-related risk factors, and assess the diagnostic performance of commonly used serological tests. The ultimate goal was to inform more effective strategies for early diagnosis and to provide a more accurate assessment of the regional infection burden.

## 2. Materials and Methods

### 2.1. Data Collection

In this retrospective study, serological test results obtained in the Medical Microbiology Laboratory between January 2020 and December 2023 were evaluated. During the study period, a total of 9819 RBT, 10,349 STA tests, and 4377 Brucellacapt tests were performed, corresponding to 24,545 serological tests in total.

The dataset included all serum samples submitted to the microbiology laboratory for suspected brucellosis testing during the study period. Therefore, no sampling strategy was applied and the analysis represents the complete tested population rather than a randomly selected sample.

After removing duplicate records belonging to the same patient, a total of 367 seropositive cases were included in the analysis. Repeated samples from the same patient, samples with insufficient volume or hemolysis, and samples with incomplete laboratory records were excluded from the study. Due to the retrospective and laboratory-based design of the study, information on classical exposure-related risk factors such as occupation, animal contact, and dietary habits was not available. Therefore, the analyses were limited to the available data, including demographic characteristics, requesting clinical departments, and temporal distribution.

### 2.2. Serological Tests

Approximately 5 mL of venous blood was collected from each patient into sterile Vacutainer tubes. The samples were centrifuged at 2000 rpm for 5 min to separate the serum, which was then aliquoted into 1 mL microtubes and stored at −20 °C until further analysis.

### 2.3. Rose Bengal Test (RBT)

All serum samples were tested using a commercial Rose Bengal Plate Test (RBPT) kit (Ser. No: 2008/1; Pendik Veterinary Control and Research Institute, Pendik, İstanbul, Türkiye). After bringing the reagents to room temperature, 30 µL of serum and antigen were mixed and rotated for 4 min. Visible agglutination was interpreted as a positive result [15]. RBT is a rapid test. It is considered suitable for screening because of its high sensitivity (>90%); however, its specificity is relatively low [16].

### 2.4. Standard Tube Agglutination Test (STA)

The STA test was performed using a commercially available inactivated *Brucella abortus* 119-3 strain antigen and saline solution in tube format (Ser. No: 2008/1; Pendik Veterinary Control and Research Institute, Pendik, Istanbul, Türkiye), in accordance with the manufacturer’s instructions. To avoid the prozone effect, sera were serially diluted from 1:20 to 1:1240. After incubation at 37 °C for 24 h, the reactions were evaluated, and the highest serum dilution showing more than 50% agglutination was recorded as the endpoint titer [17]. The STA test is considered a reference method and enables the detection of IgM, IgG, and IgA class antibodies. In the Wright STA test, a ≥4-fold increase in antibody titer between acute and convalescent serum samples obtained at a two-week interval, or a single titer of ≥1/160, is considered diagnostically significant [16]. The STA test was selected as the reference method because bacterial culture is not routinely performed in all suspected cases due to biosafety concerns, limited availability, and its time-consuming nature. Moreover, detailed and standardized clinical diagnostic criteria were not consistently accessible in this retrospective, laboratory-based dataset. Therefore, STA, which is widely used and accepted as a reference serological test in endemic settings, was considered the most appropriate comparator for evaluating the diagnostic performance of the other serological assays.

### 2.5. Brucellacapt Test

The Brucellacapt test was performed according to the manufacturer’s instructions (L-BRUCAPT; Vircell, Granada, Spain). This one-step immunocapture agglutination assay was conducted on U-bottom microtiter plates coated with anti-human immunoglobulins targeting IgG and IgA antibodies. Prior to testing, sera and reagents were brought to room temperature. After adding equal volumes (50 µL) of serum and antigen, the plates were incubated at 37 °C for 24 h. Agglutination at titers ≥ 1/160 was considered positive, whereas pellet formation indicated a negative result [18]. Brucellacapt is capable of detecting both agglutinating IgG and IgM antibodies as well as non-agglutinating IgG antibodies within a single assay [16].

### 2.6. Statistical Analysis

Data were analyzed using IBM SPSS Statistics version 25.0 (Armonk, NY, USA: IBM Corp.). Continuous variables were presented as mean ± standard deviation, whereas categorical variables were expressed as frequencies and percentages. Differences between categorical variables were analyzed using the chi-square test. Trend across years and months was evaluated using the linear-by-linear association (chi-square test for trend). The diagnostic performance of the serological tests was evaluated based on sensitivity, specificity, positive predictive value (PPV), negative predictive value (NPV), and overall accuracy, using the standard tube agglutination (STA) test as the reference method. Agreement between the tests and the STA reference test was evaluated using Cohen’s kappa coefficient. A *p*-value < 0.05 was considered statistically significant.

### 2.7. Ethical Approval

This study was approved by the Medical Research Ethics Committee of Kahramanmaraş Sütçü İmam University Faculty of Medicine (Date: 26 May 2025; Session No: 2025/18; Decision No: 03). The study was conducted in accordance with the Declaration of Helsinki.

## 3. Results

*Brucella* seropositivity was detected in 367 of the 24,545 blood samples (1.5%) analyzed during the four-year study period (2020–2023).

### 3.1. Temporal Trends in Brucellosis Positivity Rates

**Figure 1:**
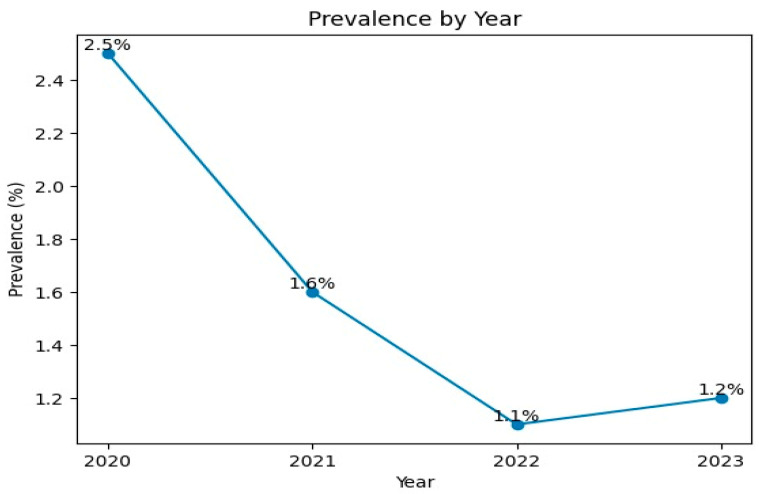
Year-to-year percentage changes (increase and decrease) in brucellosis positivity rates between 2020 and 2023.

When year-to-year changes were evaluated, it was observed that the brucellosis positivity rate was 2.5% in 2020, decreased to 1.6% in 2021 and 1.1% in 2022, and rose to 1.2% in 2023 (Figure 1).

### 3.2. Demographic, Clinical, and Laboratory Characteristics of Patients with Brucellosis

Of the 367 brucellosis cases, 57.8% were female, and 36.0% were in the 41–64 years age group. The highest positivity rate was observed in the infectious diseases department (37.1%), followed by neurology (15.3%) and pediatrics (14.7%). During the study period, 9819 RBT, 10,349 STA, and 4377 Brucellacapt tests were evaluated. Among the serological tests, Brucellacapt demonstrated the highest positivity rate (90.2%), followed by the RBT (76.2%) (Table 1).

### 3.3. Comparison of Diagnostic Tests for Brucellosis According to Demographic and Temporal Factors

High positivity rates were observed with the Brucellacapt test in both male (91.1%) and female (89.6%) patients; however, no statistically significant association was found between sex and test results. Brucellacapt yielded the highest positivity rates across all age groups, with no significant differences detected between age categories.

From 2020 to 2022, Brucellacapt demonstrated the highest positivity rates (91%, 93.3%, and 87%, respectively), whereas in 2023, RBT showed the highest rate (89.3%), representing a significant increase compared with 2020 (*p* = 0.005) (Table 2).

### 3.4. The Monthly Distribution Presented in Figure 2 Reflects Pooled Data Aggregated over the Entire Study Period (2020–2023), Rather than Month-Specific Results for Each Individual Year

**Figure 2:**
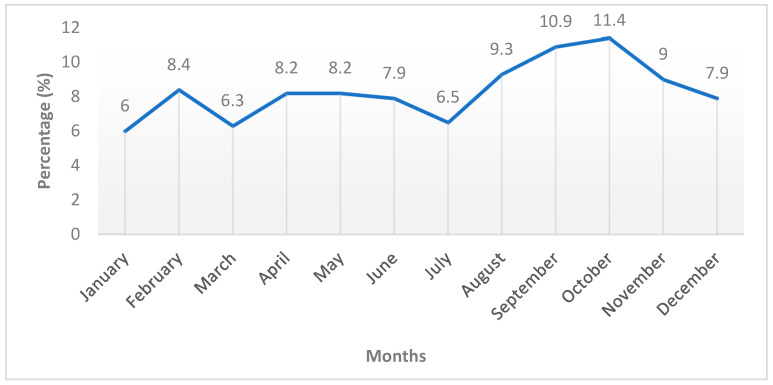
Monthly distribution of *Brucella* positivity rates over the study period (2020–2023).

Between 2021 and 2023, *Brucella* positivity peaked in October (11.4%), followed by September (10.9%) and August (9.3%). The lowest rates were observed in January (6.0%), March (6.3%), and June (6.5%). Seasonally, the highest positivity rate was recorded in autumn (31.3%) (Figure 2).

### 3.5. Diagnostic Performance of Serological Tests Using STA as the Reference Standard

Using STA as the reference standard, Brucellacapt demonstrated higher sensitivity, negative predictive value (NPV), and overall accuracy compared to the Rose Bengal test, whereas both tests exhibited low specificity. The detailed diagnostic performance parameters are summarized in Table 3. Agreement between the tests and the STA reference test was evaluated using Cohen’s kappa coefficient, which showed a slight agreement for the Rose Bengal test and a fair agreement for the Brucellacapt test.

### 3.6. Comparison of Brucellosis Diagnostic Test Results Across Clinical Departments

Table 4 presents a comparison of the RBT, STA, and Brucellacapt tests used for the diagnosis of brucellosis across different clinical departments. The positivity rate of the STA test was 37.8% in the neurology department, which was significantly lower than the rates observed in pediatrics (72.9%), infectious diseases (79.2%), internal medicine (80%), and rheumatology (84.2%) (*p* = 0.001). Similarly, the Brucellacapt test showed a significantly lower positivity rate in the neurology department (76.2%) compared to the infectious diseases department (96.6%) (*p* = 0.033). Overall, Brucellacapt demonstrated the highest positivity rates across departments, whereas STA showed significantly lower positivity in certain clinical departments.

### 3.7. Monthly Comparison of Brucellosis Diagnostic Test Results

According to the trend analysis, no significant change was observed across months. However, pairwise comparisons revealed that the positivity rate of the RBT was significantly higher in June (100%) compared with July (66.7%) (*p* = 0.029). No statistically significant differences were observed between months for the other serological tests (Table 5).

## 4. Discussion

Brucellosis remains a significant global health threat, particularly in developing countries, as well as in the Mediterranean region, the Arabian Peninsula, the Middle East, and parts of Latin America. Its incidence varies over time, between countries, and even among different regions within the same country [19]. In Türkiye, where brucellosis is considered endemic, data from the Ministry of Health indicate that the number of reported cases declined between 2008 and 2015 but began to rise thereafter, reaching an incidence of over 10 per 100,000 population in 2019 [20].

In this study, the seroprevalence of human brucellosis was low (1.5%) in a hospital-based population and was consistent with a previous hospital-based report from the same region (1%) [21]. Seroprevalence rates reported from different regions of Türkiye vary widely, ranging from 26.7% in Eastern Anatolia [22] and 15.1% in Central Anatolia [23] to 3.6% in the Mediterranean region [24] and 0.9% in the Black Sea region [19]. This variability may be attributable not only to regional factors but also to differences in sampling frames, target populations, and diagnostic methods across studies.

Similarly, brucellosis seroprevalence rates reported from other countries, including Palestine (76%) [25], Morocco (33.2%) [26], Bosnia and Herzegovina (23.3%) [27], Mexico (18.1%) [28], and Cameroon (0.98%) [29], should be interpreted within the epidemiological and methodological contexts in which those studies were conducted.

In our study, when year-to-year changes were evaluated, the brucellosis positivity rate was 2.5% in 2020, decreased to 1.6% in 2021 and 1.1% in 2022, and slightly increased to 1.2% in 2023 (Figure 1). This pattern indicates an overall decreasing trend during the study period, despite a minor fluctuation in the final year. Similar temporal variations have been reported in other studies. For example, Bouamra et al. [2], who analyzed surveillance data from Western Algeria between 2014 and 2019, reported a fluctuating but overall decreasing trend in the incidence of human brucellosis. In a study conducted in Jordan, Al-Amr et al. [7] observed a fluctuating distribution of brucellosis prevalence between 2016 and 2020, with alternating increases and decreases across the study years. Likewise, Bačić et al. [27] reported a fluctuating distribution of brucellosis cases throughout the study period in Bosnia and Herzegovina. These findings suggest that the temporal distribution of brucellosis may vary across regions depending on regional epidemiological conditions, exposure risks, surveillance systems, and access to healthcare services.

The decline in brucellosis cases may be attributed to improvements in healthcare services, increased public awareness, and strengthened disease control measures. In endemic regions such as Türkiye, national surveillance systems, livestock vaccination programs, public education on the consumption of pasteurized dairy products, and integrated One Health approaches have been implemented to reduce the burden of brucellosis [13,20].

In our study, the prevalence of brucellosis was higher among females (57.8%) than among males (42.2%) (Table 1). Although several studies have reported higher prevalence among males [19,23,27], other studies conducted both in Türkiye and abroad have indicated that brucellosis is more common in females [30,31,32,33]. Additionally, according to the 2024 report by the Turkish Ministry of Health, General Directorate of Public Health, the proportion of female brucellosis cases in Türkiye ranged from 52% to 73% [20]. This gender disparity may be attributed to the fact that females in rural areas are more frequently involved in animal care and dairy product preparation [31].

Although brucellosis can affect all age groups, its prevalence tends to increase with age and decrease after 65 years. It is particularly more common among adults aged 45–64 years [20]. In our study, the highest positivity rate was observed in the 41–64 age group, accounting for 36% of all cases (Table 1). This finding aligns with previous studies reporting higher rates of brucellosis among individuals aged 40 years and above [19,27,30,34,35]. Some studies, however, have reported higher positivity in different age groups. For example, Assafi et al. [4] reported a rate of 22.7% in the 21–30 age group, while Bouamra et al. [2] identified increased rates among individuals aged 25–44 years and older. The higher positivity rate in the 41–64 age group in our study may be attributed to greater exposure to risk factors such as involvement in animal husbandry in rural areas, consumption of raw milk and dairy products, and the cumulative risk of infection that increases with age.

In our study, no statistically significant relationship was found between test results and gender or age groups (Table 2). However, the highest positivity rate was observed with the RBT in 2023 (89.3%), showing a significant increase compared with 2020 (*p* = 0.005) (Table 2). This increase may be attributed to the major earthquake that struck the region in 2023. Post-earthquake conditions, such as poor hygiene, food safety issues, and increased consumption of unpasteurized dairy products, likely elevated *Brucella* transmission risk. Additionally, disrupted access to healthcare may have delayed diagnosis and follow-up. The significant change was observed only in RBT, possibly due to its sensitivity to acute infections, unlike Brucellacapt and STA, which reflect more chronic stages.

The current study showed that brucellosis cases peaked in October (11.4%), while the lowest prevalence was recorded in January (6%) (Figure 2). This finding is consistent with the study by Aydın et al. [14], which reported that the rise in cases beginning in spring reached its peak in late summer and early autumn. Similarly, the study by Bačić et al. [27] in Bosnia and Herzegovina partially supports our results, indicating the highest incidence in October (14.19%) and the lowest in the winter months, particularly February (1.27–5.26%).

Numerous studies have reported increased brucellosis incidence during the spring and summer seasons [2,7,30]. The seasonal discrepancy observed in our study, with a peak in October, may be attributed to local climatic variations, environmental factors, and regional public health practices. Increased livestock activity and environmental conditions during this period might have contributed to enhanced transmission of *Brucella*.

Sensitivity and specificity are fundamental metrics used to assess the accuracy of diagnostic tests. Sensitivity refers to the ability of a test to correctly identify individuals with the disease, whereas specificity indicates the ability to correctly identify those without the disease. These two metrics are often inversely related; an increase in one may lead to a decrease in the other. Moreover, the PPV and NPV provide insights into the likelihood that positive or negative test results reflect the true disease status. When considered together, these parameters offer a more comprehensive understanding of the overall reliability of a diagnostic test [36].

Serological tests are preferred for the diagnosis of brucellosis because culture is time-consuming and poses biosafety risks. Although the commonly used RBT and STA are rapid, inexpensive, and practical, each has important limitations. RBT may yield false-positive results due to cross-reactions, while STA may result in false-negative results, particularly in chronic cases [9]. However, accurate diagnosis requires tests with both high PPV to confirm the presence of disease and high NPV to reliably exclude it. The primary objective of the diagnostic process is to correctly identify true cases while minimizing false-positive results [37].

Because no single serological test provides both high sensitivity and high specificity for the diagnosis of brucellosis, the Rose Bengal test, STA, and Brucellacapt were used in combination in the present study.

The Rose Bengal test is a rapid screening method with high sensitivity, particularly in acute cases; however, due to the risk of false-positive results in endemic regions, its findings require confirmation with additional tests [15].

The standard tube agglutination test (STA) is a classical reference method that has long been included in diagnostic guidelines and generally demonstrates higher specificity; nevertheless, its sensitivity may be reduced in chronic cases and in the presence of blocking or persistent antibodies [38].

Brucellacapt, on the other hand, has been reported as a complementary serological assay with higher sensitivity than STA, particularly for the detection of persistent IgG antibodies and the identification of chronic cases [39].

Recent studies have shown that the combined use of multiple serological tests (e.g., Rose Bengal + STA + Brucellacapt or ELISA) significantly improves diagnostic accuracy compared with single-test approaches and enhances the balance between sensitivity and specificity, especially in endemic settings [1]. Therefore, by using these three tests together, we aimed to improve the detection of both acute and chronic/persistent cases while reducing false-positive and false-negative results.

In our study, the RBT and Brucellacapt tests demonstrated different diagnostic performances in terms of sensitivity, specificity, PPV, and NPV. While the RBT showed high sensitivity (81.8%), its specificity was considerably low (32.4%), indicating that although the test is effective in detecting brucellosis cases, it may frequently yield false-positive results. The lower-than-expected specificity of the Rose Bengal test observed in our study is consistent with the endemic nature of brucellosis in the study region and the presence of persistent antibody responses. In endemic areas, individuals with a history of acute brucellosis or repeated exposure may remain positive by the Rose Bengal and tube agglutination tests even years after infection, which has been reported to substantially reduce test specificity and, in particular, the positive predictive value [40]. In addition, S-LPS–based serological tests such as the Rose Bengal test may exhibit cross-reactivity with Gram-negative bacteria sharing similar LPS structures (e.g., *Yersinia enterocolitica* O:9 and *Salmonella* spp.), thereby contributing to false-positive results [41]. The PPV and NPV of the RBT were 71.6% and 46.2%, respectively. The relatively high PPV suggests that positive results are likely to reflect true infection, whereas the low NPV indicates that negative results may not reliably exclude brucellosis. Therefore, although RBT is suitable for screening purposes, confirmatory testing is recommended for accurate diagnosis (Table 3).

In our study, the sensitivity of the RBT was consistent with previous findings; however, differences were observed in specificity, PPV, and NPV values compared with the literature. Similar to our results, Zakaria [37] and Gültekin et al. [9] reported high sensitivity but relatively low specificity. Conversely, Loubet et al. [1] reported uniformly high diagnostic values. These discrepancies may be attributed to variations in brucellosis prevalence within the tested populations, differences in the gold standard methods used for comparison, the conditions under which the tests were performed, or heterogeneity in the study populations.

The Brucellacapt test detects both agglutinating and non-agglutinating antibodies [12]. In our study, the sensitivity of the Brucellacapt test was found to be high (94.7%), indicating that it is effective in detecting brucellosis cases. The higher positivity rate observed with the Brucellacapt test may be explained by the intracellular persistence and immune evasion strategies of *Brucella* spp., which contribute to prolonged antibody responses in chronic infection [8]. However, its low specificity (27.6%) suggests a high probability of false-positive results among individuals without the disease. The low specificity observed for the Brucellacapt test in our study may similarly be explained by previous infections in endemic regions and persistent IgG antibody responses. As an immunocapture/agglutination-based assay, Brucellacapt is able to readily detect incomplete or persistent antibodies, particularly in chronic cases and in individuals with prior exposure, which may result in false-positive findings that are not consistent with clinically active disease [15]. Several studies have reported that when used alone, Brucellacapt shows poor correlation with other serological tests and frequently yields inconclusive results [1,42]. Therefore, it is emphasized that the use of Brucellacapt within combined diagnostic algorithms together with the Rose Bengal test and/or ELISA provides higher diagnostic accuracy compared with its use as a standalone test, by reducing false-positive results and improving specificity [1,42]. The PPV was 71.8% and the NPV was 72.7%, indicating that positive results are strongly associated with actual disease, while negative results are relatively reliable for excluding brucellosis (Table 3).

Therefore, the Brucellacapt test is useful in diagnosis due to its high sensitivity, but positive results should be supported by confirmatory tests because of its low specificity. When the findings of the Brucellacapt test were compared with the literature, notable discrepancies emerged. Loubet et al. [1] reported a sensitivity of 82.4%, specificity of 99.3%, PPV of 84.1%, and NPV of 99.2% for Brucellacapt. Casanova et al. [18] reported a sensitivity of 91.6%, specificity of 95.9%, PPV of 99.2%, and NPV of 67.1%. Xu et al. [12] reported a sensitivity of 88.37%, specificity of 100%, PPV of 100%, and NPV of 86.3%. Uysal et al. [11] reported these values as 71.4%, 25%, 41.6%, and 53.8%, respectively, in acute cases. The differences between our findings and those reported in the literature may be attributed to factors such as the stage of *Brucella* infection, the immune status of the patients, the *Brucella* strains used, variations in test implementation methods, and differences in reference standards. These factors can cause variability in the sensitivity and specificity of serological tests, leading to discrepancies in results.

In our study, statistical analysis revealed a significant association between the results of the STA and Brucellacapt tests and the clinical departments (*p* < 0.05) (Table 4). These differences may be attributed to the diversity of clinical presentations and the timing of patients’ hospital admissions. Therefore, the results of diagnostic tests should be interpreted in conjunction with the clinical context.

In our study, when the distribution of test results across months was analyzed, a statistically significant difference was observed only for the RBT test. Specifically, the positivity rate in June (100%) was significantly higher compared with July (66.7%) (*p* = 0.029) (Table 5). This finding may be attributed to the high sensitivity of RBT, which enables it to detect acute or newly emerging infections at earlier stages. In contrast, confirmatory tests such as STA and Brucellacapt are more specific and typically yield positive results in later stages of infection or in the presence of more persistent antibody responses. Therefore, it is expected that RBT is more responsive to seasonal fluctuations.

The main limitation of this study is its retrospective, laboratory-based design, which relied on routine serological records and did not allow consistent retrieval of detailed clinical correlation data, including clinical presentation, disease severity, or treatment outcomes. In addition, information on classical exposure-related risk factors such as occupation, animal contact, and dietary habits was unavailable. Furthermore, because the data were obtained from routine laboratory records of a tertiary care hospital, the study may be subject to potential selection bias. Only patients who were clinically suspected of brucellosis and referred for laboratory testing were included, and therefore the findings may not fully represent the general population. Accordingly, the reported seroprevalence should be interpreted as a hospital-based estimate rather than a population-based prevalence. Another limitation is the absence of culture or molecular confirmation of the diagnosis, as well as the single-center nature of the study, which may restrict the generalizability of the findings. Future prospective, multicenter studies incorporating standardized clinical data, detailed exposure histories, and combined serological and molecular diagnostic approaches are needed to better define disease burden and improve diagnostic strategies in endemic regions.

## 5. Conclusions

This study demonstrated that the seroprevalence of brucellosis in a tertiary care hospital was relatively low, although the disease remains an important public health concern in endemic regions. The annual distribution of cases showed an overall decreasing trend during the study period. The highest positivity rates were observed among patients admitted to the infectious diseases department and in the middle-aged group. Among the diagnostic methods evaluated, the Brucellacapt test demonstrated high sensitivity and served as a valuable complementary diagnostic tool in the evaluation of brucellosis. However, its low specificity highlights the need for careful interpretation of positive results and supports its use in combination with other serological tests to improve diagnostic accuracy. Considering the seasonal, regional, and departmental variations observed in this study, a combined testing approach may enhance overall diagnostic performance.

## Figures and Tables

**Table 1:** Demographic characteristics, clinical departments, and laboratory test results of patients diagnosed with brucellosis.

Variable		Frequency	Percent
Gender	Male	155	42.2
	Female	212	57.8
Age groups	≤17 years and under	47	12.8
	18–40 years	102	27.8
	41–64 years	132	36.0
	≥65 years and over	86	23.4
Clinical Departments	Infection	136	37.1
	Pediatrics	54	14.7
	Gastroenterology	19	5.2
	Neurology	56	15.3
	Neurosurgery	28	7.6
	Rheumatology	30	8.2
	Internal medicine	11	3.0
	Other (nephrology, orthopedics and traumatology, physical medicine and rehabilitation, urology, etc.)	33	9.0
Rose Bengal test	Positive	250	76.2
	Negative	78	23.8
Standard tube agglutination test	Positive	178	68.7
	Negative	81	31.3
Brucellacapt test	Positive	212	90.2
	Negative	23	9.8

**Table 2:** Comparison of the diagnostic tests used for brucellosis according to age, gender, and year.

**Variable**	**Tests**								
	**Rose Bengal**		***p*-Value**	**Standard Tube Agglutination**		***p*-Value**	**Brucellacapt**		***p*-Value**
**Gender**	**Positive** **n (%)**	**Negative** **n (%)**		**Positive** **n (%)**	**Negative** **n (%)**		**Positive** **n (%)**	**Negative** **n (%)**	
Male	104 (75.9)	33 (24.1)	0.912 (χ^2^ = 0.012)	83 (72.2)	32 (27.8)	0.285 (χ^2^ = 1.144)	92 (91.1)	9 (8.9)	0.695 (χ^2^ = 0.154)
Female	146 (76.4)	45 (23.6)		95 (66.0)	49 (34.0)		120 (89.6)	14 (10.4)	
**Age groups**	**Positive**	**Negative**	***p*-value**	**Positive**	**Negative**	***p*-value**	**Positive**	**Negative**	***p*-value**
Ages 17 and under	26 (76.5)	8 (%23.5)	0.585 (χ^2^ = 1.941)	31 (72.1)	12 (27.9)	0.119 (χ^2^ = 5.856)	35 (92.1)	3 (7.9)	0.47 (χ^2^ = 2.531)
18–40 age group	64 (72.7)	24 (%27.3)		58 (78.4)	16 (21.6)		65 (92.9)	5 (7.1)	
41–64 age group	101 (80.2)	25 (%19.8)		55 (62.5)	33 (37.5)		69 (90.8)	7 (9.2)	
Ages 65 and over	59 (73.8)	21 (26.3)		34 (63.0)	20 (37.0)		43 (84.3)	8 (15.7)	
**Years**	**Positive**	**Negative**	***p*** **-value**	**Positive**	**Negative**	***p*** **-value**	**Positive**	**Negative**	***p*** **-value**
2020	57 (64.8)	31 (35.2)	0.0001 * (χ^2^ = 12.663)	50 (72.5)	19 (27.5)	0.736 (χ^2^ = 0.114)	61 (91.0)	6 (9.0)	0.333 (χ^2^ = 0.937)
2021	71 (74.7)	24 (25.3)		56 (65.9)	29 (34.1)		70 (93.3)	5 (6.7)	
2022	72 (80.9)	17 (19.1)		43 (68.3)	20 (31.7)		47 (87.0)	7 (13.0)	
2023	50 (89.3)	6 (10.7)		29 (69.0)	13 (31.0)		34 (87.2)	5 (12.8)	

* *p*-value < 0.05 statistically significant difference.

**Table 3:** Evaluation of the diagnostic performance of serological tests using STA as the reference standard.

Tests		STA		Total						
		Positive	Negative		Sensitivity	Specificity	PPV	NPV	Total Accuracy	Kappa (*p*)
Rose Bengal	Positive	126	50	176	81.8	32.4	71.6	46.2	65.8	0.154 (0.016 *)
	Negative	28	24	52						
Total		154	74	228						
Brucellacapt	Positive	107	42	149	94.7	27.6	71.8	72.7	71.9	0.262 (0.0001 *)
	Negative	6	16	22						
Total		113	58	171						

PPV: positive predictive value; NPV: negative predictive value; STA: Standard Tube agglutination. * *p*-value < 0.05 statistically significant difference.

**Table 4:** Clinical department–wise comparison of diagnostic tests for brucellosis.

Clinical Departments	Rose Bengal		Standard Tube Agglutination		Brucellacapt	
	Positive n (%)	Negative n (%)	Positive n (%)	Negative n (%)	Positive n (%)	Negative n (%)
Infection	99 (79.8)	25 (20.2)	76 (79.2)	20 (20.8)	85 (96.6)	3 (3.4)
Pediatrics	26 (74.3)	9 (25.7)	35 (72.9)	13 (27.1)	46 (95.8)	2 (4.2)
Gastroenterology	12 (66.7)	6 (33.3)	8 (66.7)	4 (33.3)	11 (84.6)	2 (15.4)
Neurology	44 (78.6)	12 (21.4)	14 (37.8)	23 (62.2)	16 (76.2)	5 (23.8)
Neurosurgery	21 (75.0)	7 (25.0)	13 (50.0)	13 (50.0)	20 (83.3)	4 (16.7)
Rheumatology	21 (72.4)	8 (27.6)	16 (84.2)	3 (15.8)	11 (78.6)	3 (21.4)
Internal medicine	4 (50.0)	4 (50.0)	4 (80.0)	1 (20.0)	10 (90.9)	1 (9.1)
Other	23 (76.7)	7 (23.3)	12 (75.0)	4 (25.0)	13 (81.3)	3 (18.8)
*p*-value	0.68 (χ^2^ = 4.837)		0.001 * (χ^2^ = 27.25)		0.033 * (χ^2^ = 15.249)	

* *p*-value < 0.05 statistically significant difference.

**Table 5:** Month-wise comparison of diagnostic tests for brucellosis.

Months	Rose Bengal		Tube Agglutination		Brucellacapt	
	Positive n (%)	Negative n (%)	Positive n (%)	Negative n (%)	Positive n (%)	Negative n (%)
January	15 (78.9)	4 (21.1)	8 (66.7)	4 (33.3)	13 (100.0)	0 (0.0)
February	19 (67.9)	9 (32.1)	10 (55.6)	8 (44.4)	23 (92)	2 (8.0)
March	14 (70.0)	6 (30.0)	10 (71.4)	4 (28.6)	17 (94.4)	1 (5.6)
April	19 (67.9)	9 (32.1)	8 (47.1)	9 (52.9)	25 (96.2)	1 (3.8)
May	23 (85.2)	4 (14.8)	16 (72.7)	6 (27.3)	14 (87.5)	2 (12.5)
June	26 (100.0)	0 (0.0)	20 (76.9)	6 (23.1)	10 (76.9)	3 (23.1)
July	14 (66.7)	7 (33.3)	12 (60.0)	8 (40.0)	9 (81.8)	2 (18.2)
August	21 (72.4)	8 (27.6)	20 (76.9)	6 (23.1)	18 (81.8)	4 (18.2)
September	28 (75.7)	9 (24.3)	18 (64.3)	10 (35.7)	28 (93.3)	2 (6.7)
October	26 (68.4)	12 (31.6)	25 (75.8)	8 (24.2)	28 (93.3)	2 (6.7)
November	24 (82.8)	5 (17.2)	17 (73.9)	6 (26.1)	20 (90.9)	2 (9.1)
December	21 (80.8)	5 (19.2)	14 (70.0)	6 (30.0)	7 (77.8)	2 (22.2)
*p*-value	0.626 (χ^2^ = 0.238)		0.188 (χ^2^ = 1.736)		0.264 (χ^2^ = 1.247)	

## Data Availability

The original contributions presented in this study are included in the article. For further inquiries, please contact the corresponding author.

## Abbreviations

The following abbreviations are used in this manuscript:

WHO	World Health Organization
RBT	Rose Bengal test
STA	Standard tube agglutination test
PPV	Positive predictive value
NPV	Negative predictive value
